# Genome-wide association analysis unveils candidate genes and loci associated with aplasia cutis congenita in pigs

**DOI:** 10.1186/s12864-023-09803-6

**Published:** 2023-11-21

**Authors:** Fuchen Zhou, Shenghui Wang, Haojun Qin, Haiyu Zeng, Jian Ye, Jie Yang, Gengyuan Cai, Zhenfang Wu, Zebin Zhang

**Affiliations:** 1 College of Animal Science and National Engineering Research Center for Breeding Swine Industry, South China Agricultural University, Guangdong, 510642 P.R. China; 2 Guangdong Wens Breeding Swine Technology Co., Ltd, Guangdong, 527400 P.R. China

**Keywords:** Aplasia cutis congenita, Congenital disorder, Genome-wide association study, Pig, Candidate gene

## Abstract

**Background:**

Aplasia cutis congenita (ACC) is a rare genetic disorder characterized by the localized or widespread absence of skin in humans and animals. Individuals with ACC may experience developmental abnormalities in the skeletal and muscular systems, as well as potential complications. Localized and isolated cases of ACC can be treated through surgical and medical interventions, while extensive cases of ACC may result in neonatal mortality. The presence of ACC in pigs has implications for animal welfare. It contributes to an elevated mortality rate among piglets at birth, leading to substantial economic losses in the pig farming industry. In order to elucidate candidate genetic loci associated with ACC, we performed a Genome-Wide Association Study analysis on 216 Duroc pigs. The primary goal of this study was to identify candidate genes that associated with ACC.

**Results:**

This study identified nine significant SNPs associated with ACC. Further analysis revealed the presence of two quantitative trait loci, 483 kb (5:18,196,971–18,680,098) on SSC 5 and 159 kb (13:20,713,440–207294431 bp) on SSC13. By annotating candidate genes within a 1 Mb region surrounding the significant SNPs, a total of 11 candidate genes were identified on SSC5 and SSC13, including *KRT71*, *KRT1*, *KRT4*, *ITGB7*, *CSAD*, *RARG*, *SP7*, *PFKL*, *TRPM2*, *SUMO3*, and *TSPEAR*.

**Conclusions:**

The results of this study further elucidate the potential mechanisms underlying and genetic architecture of ACC and identify reliable candidate genes. These results lay the foundation for treating and understanding ACC in humans.

**Supplementary Information:**

The online version contains supplementary material available at 10.1186/s12864-023-09803-6.

## Background

Aplasia cutis congenita (ACC) is a rare congenital disorder characterized by the localized or widespread absence of skin in humans and animals [1, 2]. The lesions can range from a few millimeters to more than 10 cm in diameter. Some defects can have a membranous covering that can be filled with fluid, giving it a bullous appearance [3]. In 1826, Campbell initially documented cases of skin aplasia on the scalp of infants [4], with the potential for involvement in other body regions and leading to neonatal mortality [5]. In human research, studies on ACC have primarily been confined to case analyses of individual patients, with a lack of in-depth investigation into the disease [6–8]. This may be attributed to the rarity of the condition, making it difficult to gather a large number of samples. Coi et al. [3] conducted a study on ACC cases in Europe from 1998 to 2017 and found a prevalence rate of 5.2 cases per 100,000 live births among newborns. The presence of ACC in pigs has implications for animal welfare. It contributes to an elevated mortality rate among piglets at birth, leading to substantial economic losses in the pig farming industry. In 2006, Odile et al. [9] reported that ACC commonly occurs in the tail, back, thighs, and abdomen of piglets. The affected areas exhibit red lesions with a depressed appearance, lacking skin and hair coverage in the surrounding regions. ACC may cooccur with developmental anomalies such as, muscular and skeletal abnormalities [10], while its precise etiology and pathogenesis remain elusive. Genetic factors have been proposed as possible contributors, and case studies have identified instances with both autosomal dominant and recessive inheritance patterns [9, 11]. Nonetheless, the genetic underpinnings of ACC have yet to be fully elucidated, highlighting the need for further research in this area.

Since the successful completion of the Human Genome Project in 2003, remarkable advancements in genome sequencing technology have led to a substantial reduction in sequencing costs [12]. These breakthroughs have paved the way for a comprehensive understanding of the phenotypic variations and disease occurrences resulting from genomic variations [12]. In 2005, Klein et al. pioneered the application of Genome-Wide Association Study (GWAS) in investigating human macular degeneration [13], marking the beginning of GWAS as a powerful tool for unraveling candidate genes associated with both human diseases [14, 15] and economically significant traits [16, 17]in agriculture. In our previous research, we utilized GWAS to identify potential genes related to several crucial economic traits in pigs, including lean meat percentage [18], teat number [19], and intramuscular fat content [20].

Due to the low probability of ACC occurrence, it took us 5 years (2017–2021) to collect a total of 116 ACC-affected piglets (58 from American Duroc population and 48 from Canadian Duroc population) and 100 healthy piglets (50 from American Duroc population and 50 Canadian Duroc population). Subsequently, we conducted a genome-wide association analysis on a total of 216 Duroc piglets to elucidate candidate genetic loci associated with ACC. The primary goal of this study was to identify candidate genes that contribute to the development of ACC, thus providing valuable insights into the genetic architecture underlying skin aplasia. By leveraging the pig as a model organism, our research aimed to unravel the fundamental mechanisms governing skin development disorders, ultimately offering valuable implications for treating of related human diseases.

## Result

### Phenotype and SNP genotyping

**Fig. 1:**
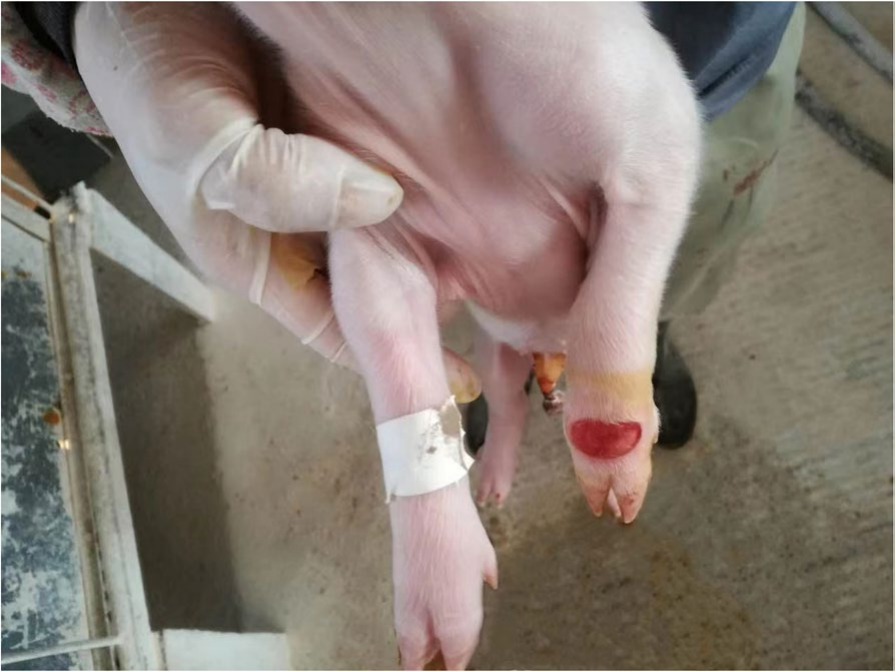
Illustration of a piglet with ACC on the left forehoof

**Fig. 2:**
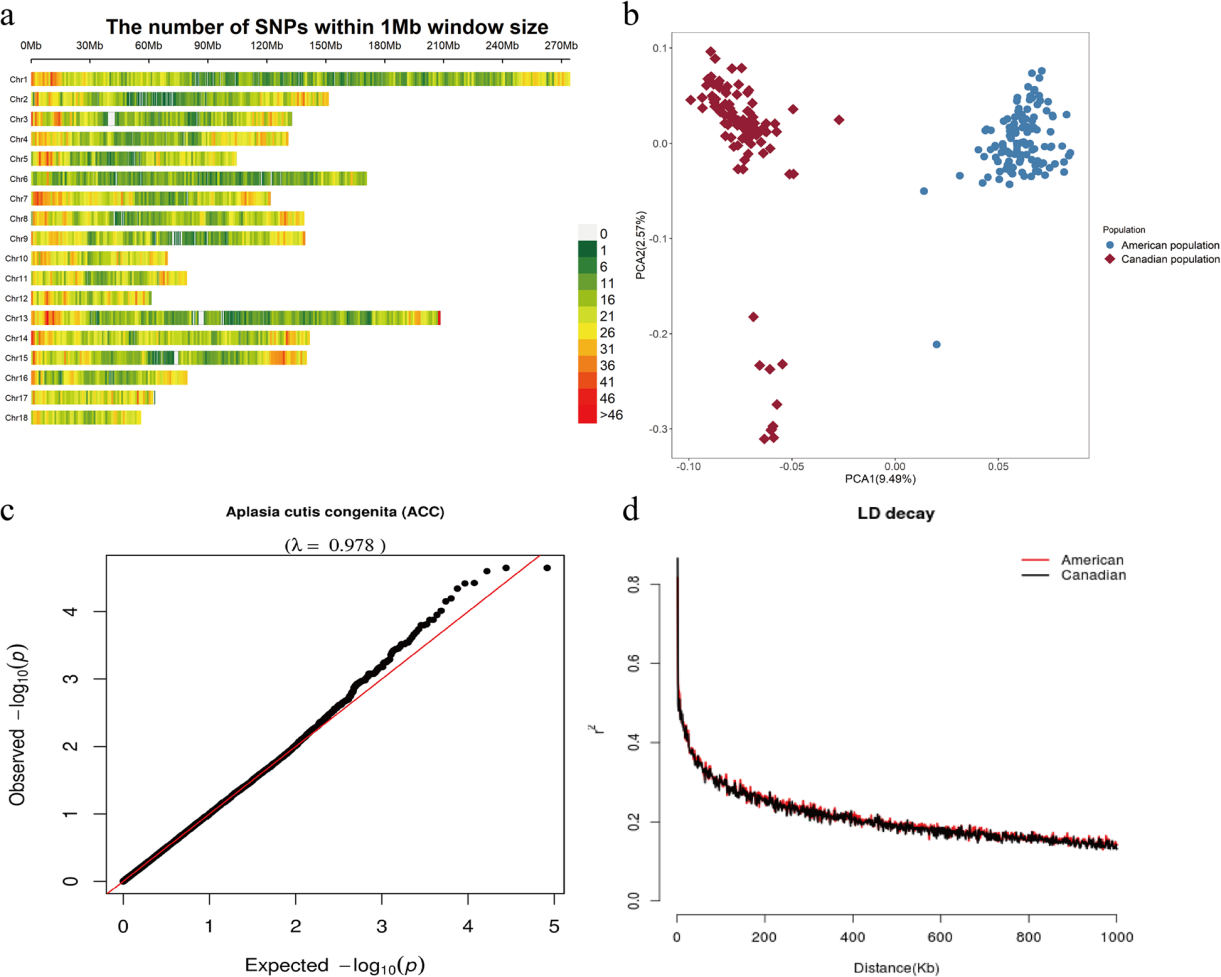
Distribution of SNPs across Chromosomes after quality control (**a**), population structure for two Duroc populations (**b**), Quantile–quantile (Q–Q) plots of genome-wide association studies for ACC in the two populations (**c**), and LD decay across the whole genome of the association population (**d**). For **a**, the Y-axis values are autosomes and X-axis are chromosome position in Mb. For **b**, the PCA1 present first principal component, PCA2 is second principal component. The first two PCs derived from the genomic kinship matrix were extracted to assess the population structure. The plot **c** shows the observed versus expected negative log10 *P*-value. In plot 4, the red and black colors represent LD decay in the American and Canadian Duroc populations, respectively

ACC can occur in various parts of piglets' bodies (Additional file 1: Fig S1). Figure 1 illustrates a case of ACC on the left forehoof of a piglet, characterized by a well-defined border, a bright red base, a relatively small lesion area, and no apparent discharge. After DNA extraction from tail tissue samples of the 216 piglets, we performed genotyping using the GenoBaits Porcine 50 K single nucleotide polymorphism (SNP) Chip. The quality of genotyping of the 216 Duroc pigs was examined using PLINK v1.9. The distribution and visualization of the SNP dataset across chromosomes are summarized in Fig. 2a and Additional file 2: Table S1. These SNPs were roughly proportionally distributed on all 18 chromosomes of pigs, with the longest chromosome having the most significant number of SNPs. As shown in Fig. 2a, we observed a higher density of SNP distribution towards the ends of the chromosomes. This pattern may be attributed to the limitations of the sequencing technology, which is consistent with the observed distribution and provides further evidence for the reliability of our sequencing results. Additionally, *Sus scrofa* chromosome (SSC) 6 exhibited the most uniform distribution of SNPs.

### Population structure and LD decay

Considering the involvement of two Duroc populations in this study, we were concerned about the potential presence of population stratification. Therefore, we conducted a Principal component analysis (PCA). As presented in Fig. 2b, the PCA plot showed that the American and Canadian Duroc pigs did not coincide, indicating that these two populations have different genetic backgrounds. In addition to incorporating the genetic relationship matrix as a covariate in the Mixed linear model (MLM) model, we included the first five principal components from PCA as covariates to adjust for population structure. After adjust, Q-Q plots with genomic inflation factors were generated to evaluate the influence of population structure on single-locus GWAS (Fig. 2c). Notably, no significant inflation of test statistics was observed in the admixed population (American and Canadian Duroc population), indicating that the results were not biased by population stratification. The average Linkage disequilibrium (LD) decay distances of the American and Canadian Duroc pig populations (*r*^*2*^ = 0.2) were approximately 500 kb (Fig. 2d), suggesting a comparable LD level between the two populations. Consequently, we treated the American and Canadian Duroc populations as a unified group for further analysis.

### Single-locus GWAS results

**Fig. 3:**
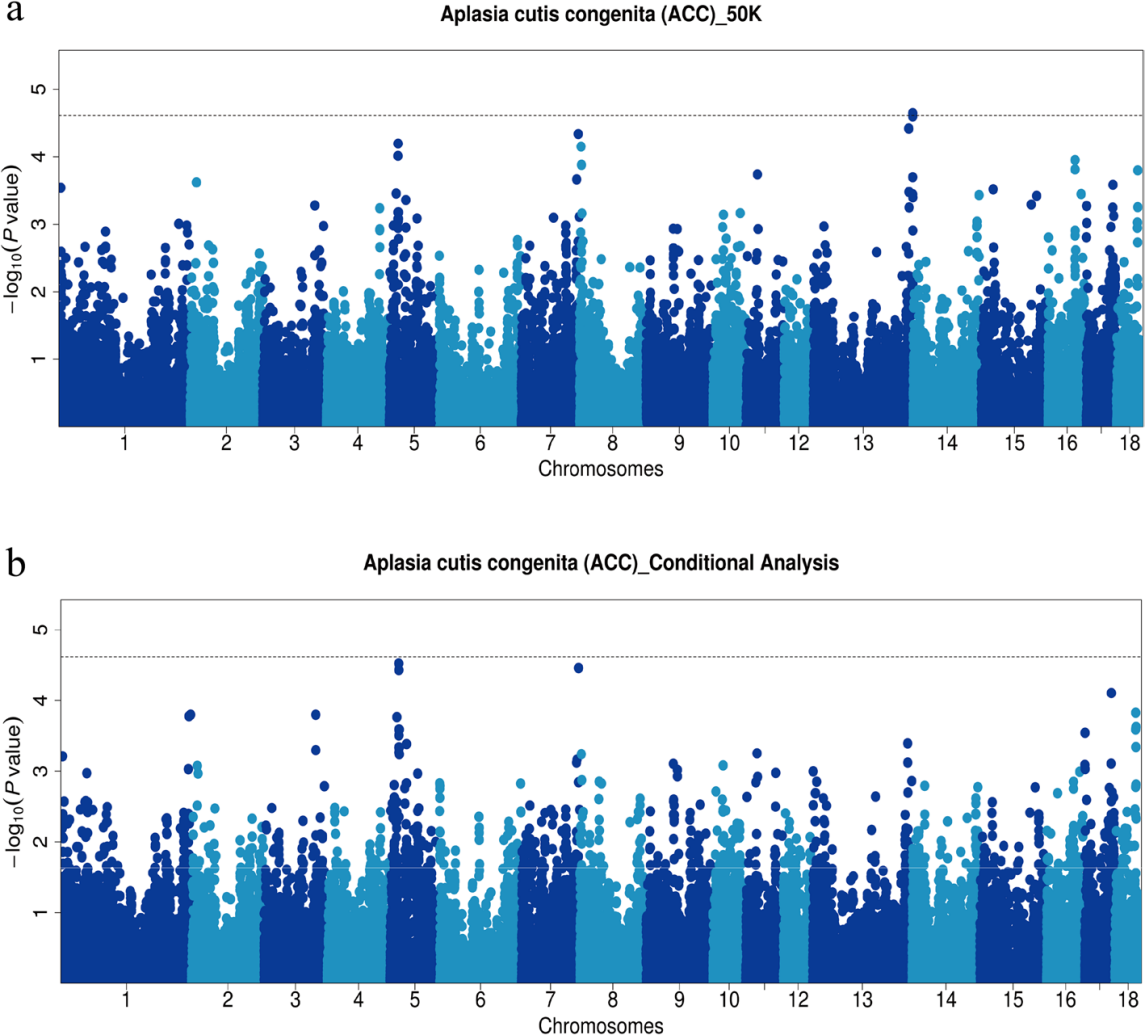
Manhattan plots of GWAS (**a**) and conditional analysis (**b**) for ACC in 216 pigs using 50 K chip data. The x-axis represents the chromosomes, and the y-axis represents the -log10(*P*-value). The dashed lines indicate the suggestive thresholds for ACC (*P* = 2.42E-5)

**Fig. 4:**
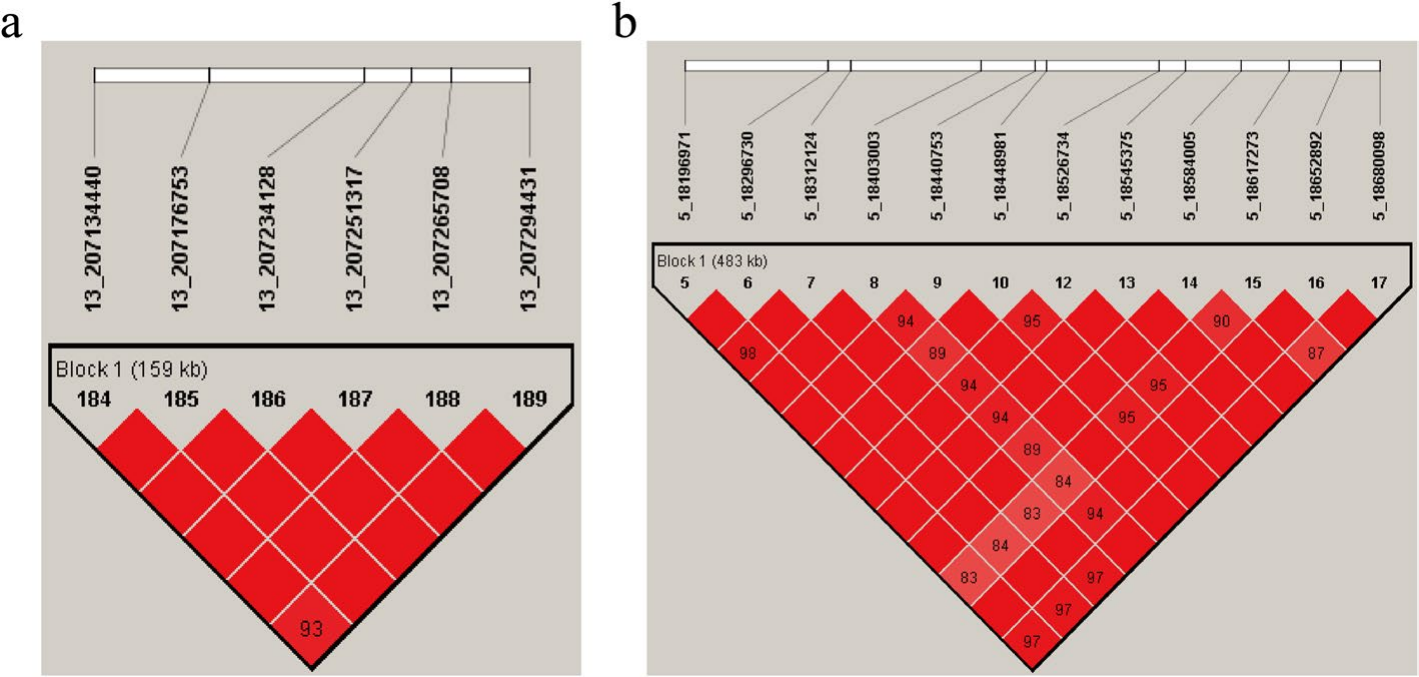
LD blocks in the significant region on SSC13 (**a**) and SSC5 (**b**). LD blocks are marked with triangles. Values in boxes are LD (r^2^) between SNP pairs and the boxes are colored according to the standard Haploview color scheme

After analyzing the quality-controlled SNPs and phenotype data using the MLM model in GEMMA software [21], significant SNPs detected through 50 K chips GWAS are presented in Table 1 and Fig. 3a. A total of 2 SNPs surpassed the suggestive significance threshold (*P* = 2.42E-5) and were identified as associated with ACC. Two SNPs (13_207176753 (*P* = 2.25E-5) and13_207265708 (*P* = 2.25E-5)) on SSC13 were found to be the lead SNPs. They are in complete LD and located within a 159 kb haplotype block (13:20,713,440–207294431 bp), which suggests that mutations near the potential quantitative trait locus (QTL) may have essential effect on ACC (Fig. 4a). Furthermore, GWAS conducted by imputed data revealed nine and six significant SNPs on SSC 13 and 10, respectively. Among these, the top SNP on SSC 13 was identified as 13_206783027_C (*P* = 2.49E-7). For more comprehensive information, please consult Fig. S2 and Table S2.

**Table 1:** Significant and strong LD SNPs associated with ACC in Duroc pigs identified by 50 K chips GWAS

SSC^a^	SNP ID	Position (bp)^b^	MAF	*P*-value	Nearest gene	Distance (bp)^c^
5	5_18403003	18,403,003	0.051	9.72E-5	*CSAD*	within
5	5_18617273	18,617,273	0.051	6.37E-5	*SP1*	within
13	13_207176753	207,176,753	0.051	2.25E-5	*PFKL*	within
13	13_207265708	207,265,708	0.051	2.25E-5	*TRPM2*	726
13	13_207251317	207,251,317	0.049	2.52E-5	*TRPM2*	within

^a^Sus scrofa chromosome

^b^SNP position in Ensembl

^c^The SNP located upstream/downstream of the nearest gene

**Fig. 5:**
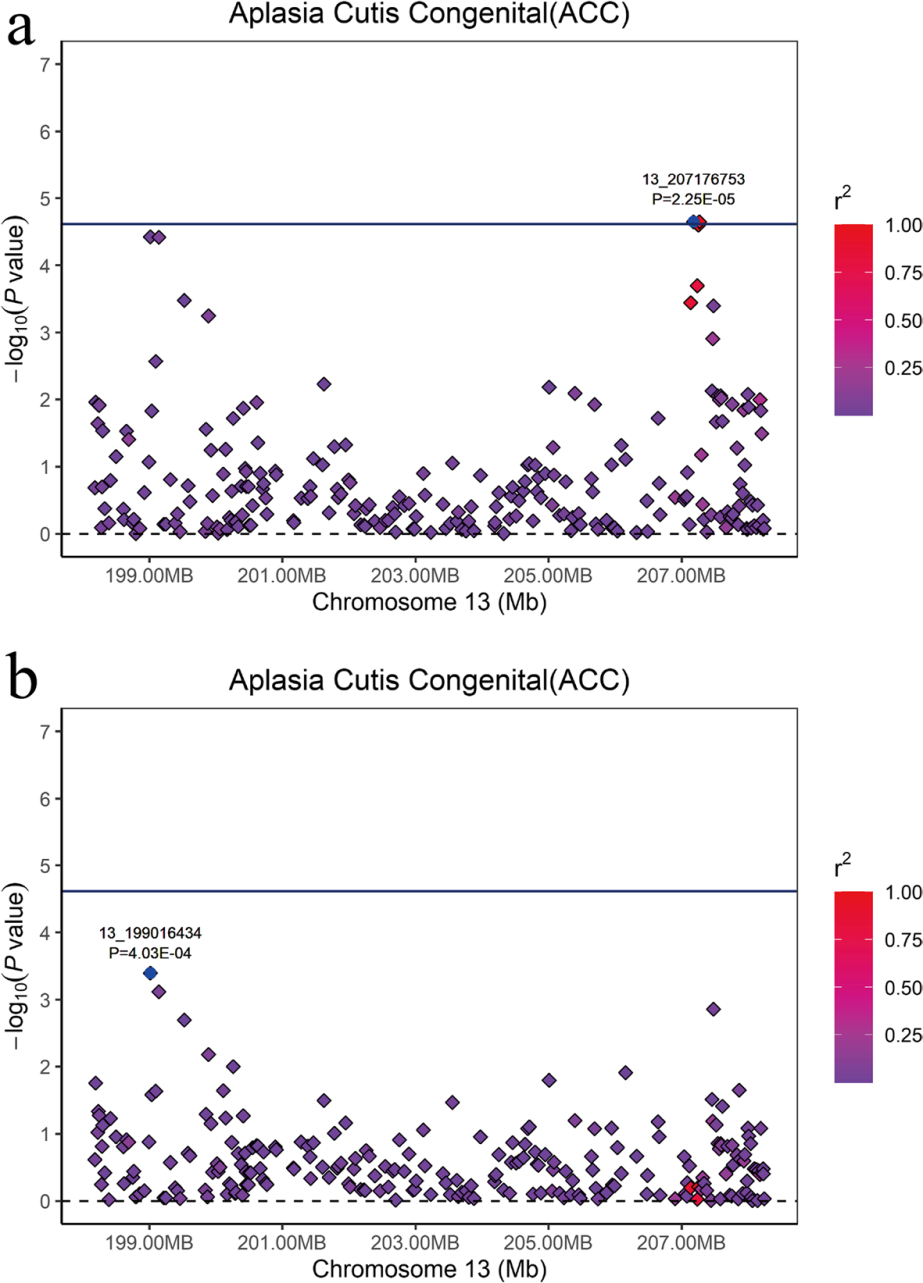
Regional association plot of the lead SNP (13_207176753) associated with ACC at SSC13. For **a** and **b** plots, the -log10(*P*-value) of SNP (Y-axis) are presented according to their chromosomal position (X-axis) on SSC13. The lead SNP of GWAS is denoted by large blue square. Other SNPs are represented by colored rhombi according to the target SNP with which they were in strongest LD. The plots **a** and** b** show the association results for ACC before and after conditional analysis on lead SNP (13_207176753)

To further investigate, we conducted a conditional analysis by fitting the lead SNP (13_207176753) as a covariate into the MLM using GEMMA software (Fig. 3b). The signal peak on SSC13, where the lead SNP is located, disappeared, and the *P*-value of the associated SNPs all dropped below the threshold line (Fig. 5a and b). This indicates a strong LD among these SNPs, indicating a significant influence of the lead SNP on ACC. Surprisingly, the signal peak on SSC5 became even more pronounced, implying the presence of a potential QTL associated with ACC (Fig. 3b). Furthermore, haplotype analysis revealed strong linkage disequilibrium (LD) between SNPs 5_18403003 (*P* = 9.72E-5) and 5_18617273 (*P* = 6.37E-5), which are located within a 483 kb haplotype block (5:18,196,971–18,680,098) (Fig. 4b). This implies that mutations in the vicinity of this region may also have substantive implications for ACC. Finally, to assess the impact of population stratification on our analysis results, we conducted GWAS using logistic mixed model with the GMMAT R package. The results were similar to those of the MLM model and showed strong signal on SSC 13. This further validates the scientific rigor of our use of the MLM model for binary trait GWAS (Additional file 5: Fig S3).

### Candidate genes search and functional annotation

**Fig. 6:**
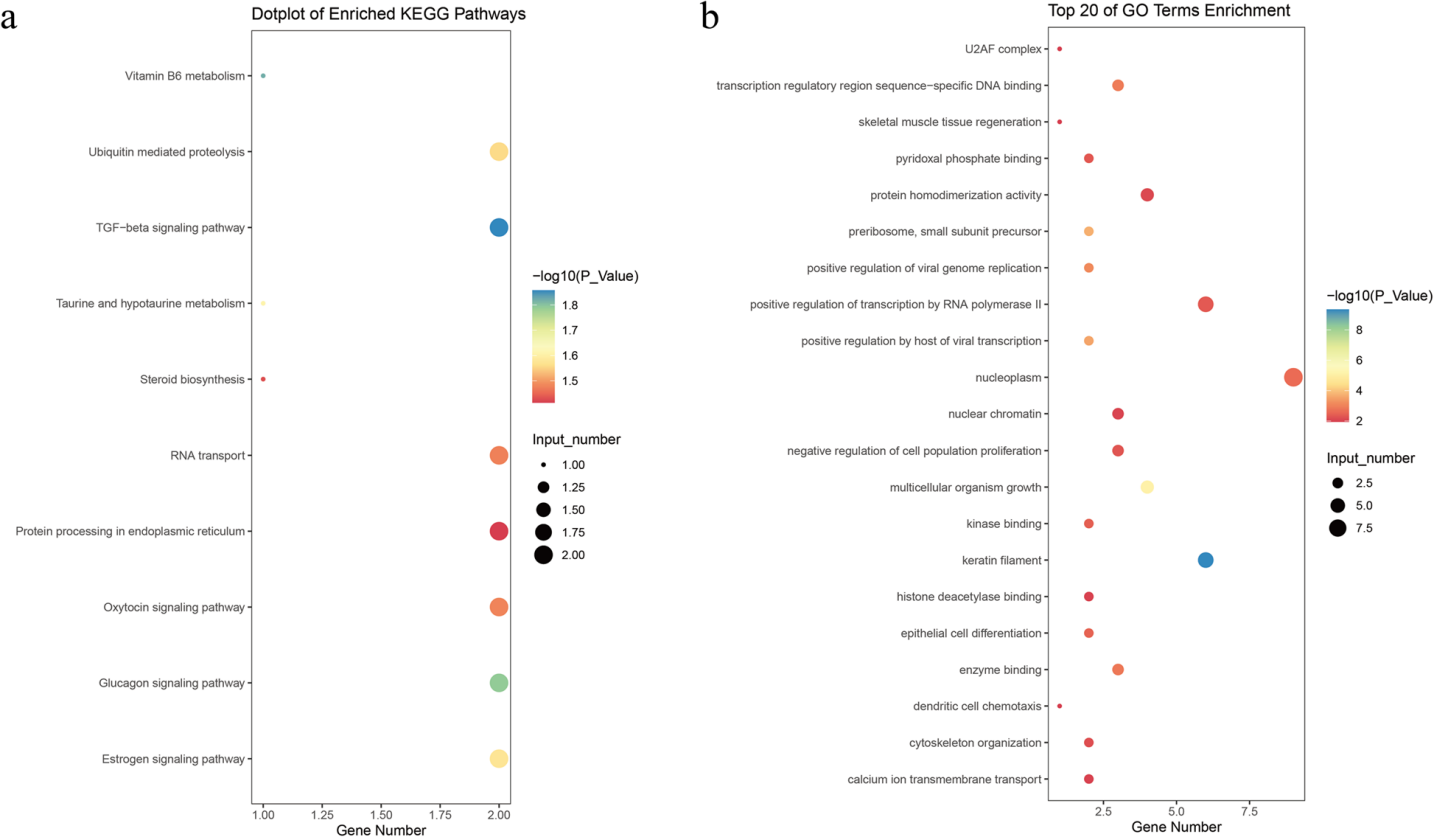
Significant KEGG pathways and GO terms associated with ACC (*P* < 0.05). The plot **a** represents the KEGG PATHWAY of the biological process for protein-coding genes within a 1 Mb region centered on the significant SNPs. The plot **b** shows the top 20 terms of the GO enrichment

**Fig. 7:**
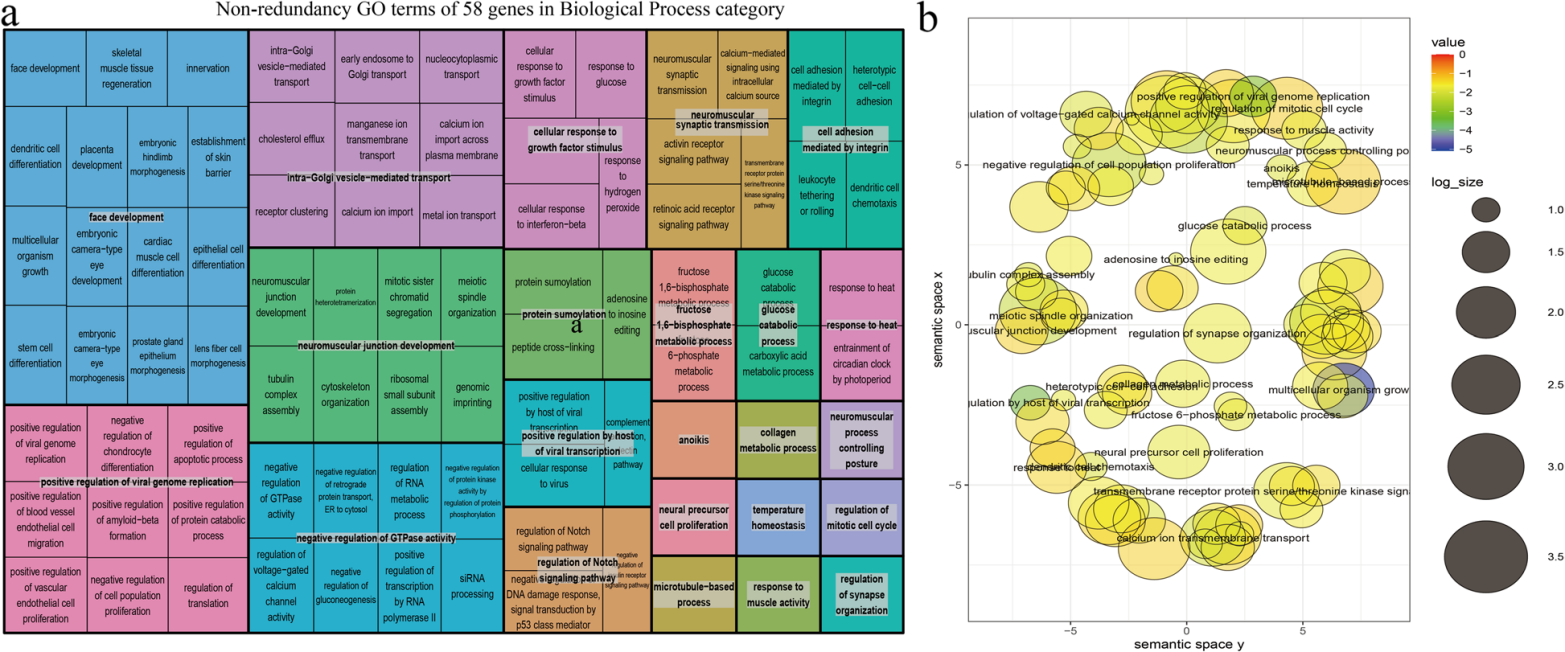
Non-redundancy GO terms of ACC-related genes in Biological Process category. The plot **a** shows the "TreeMap" view of REVIGO. Each rectangle represents a representative cluster. These representatives are combined into "superclusters," representing loosely related terms and visualized using different colors. The size of the rectangles is adjusted to reflect the *P*-value and frequency of the GO term in the Mus musculus GOA database. The plot b displays the scatter plot of representative clusters. The log size indicates the frequency of the GO term in the Mus musculus GOA database, with larger sizes indicating more common terms. The numerical value represents the -log10(*P*-value), with colors ranging from red to blue indicating increasing significance

Among the identified five SNPs on 50 K chips GWAS, four SNPs were found to be located within five genes: cysteine sulfinic acid decarboxylase (*CSAD*), Sp1 transcription factor (*SP1*), phosphofructokinase, liver type (*PFKL*), transient receptor potential cation channel subfamily M member 2 (*TRMP2*), respectively (Table 1). Based on the LD decay distance observed in the population used in this study, we observed that LD (*r*^*2*^) dropped below 0.2 after a distance of approximately 500 kb (Fig. 2d). This indicates that the causal mutations and genes may be located within a region spanning 500 kb upstream and downstream of the identified GWAS signals. To further understand the GWAS results, a total of 58 protein-coding genes within a 1 Mb region centered on the five SNPs (50 K chips GWAS) and 15 SNPs (imputed GWAS) were annotated. Additionally, we identified seven genes from the imputed GWAS that overlap with those discovered in the 50 K chip data, further underscoring the accuracy of the 50 K chip GWAS results (Additional file 6: Table S3). Our pathway enrichment analysis revealed several significantly enriched Kyoto Encyclopedia of Genes and Genomes (KEGG) and Gene Ontology (GO) terms related to ACC, including cellular cytoskeleton organization and biological development, such as skeletal muscle tissue regeneration, prostate gland epithelium morphogenesis, establishment of skin barrier, face development (Fig. 6a and b, Additional file 7: Table S4). After conducting non-redundant GO analysis on all GO terms that exceeded the threshold using the REVIGO website, a total of 87 GO terms were clustered. (Fig. 7a and b). One pathway stood out as particularly intriguing, as it is related to the process of skin barrier (GO:0061436). Subsequently, we employed the GeneCards, Mouse Genome Informatics databases, and conducted an extensive literature review to explore the functional roles of the identified genes. As a result, we identified a total of 11 candidate genes with potential relevance to ACC. These genes, namely *KRT71*, *KRT1*, *KRT4*, *ITGB7*, *CSAD*, *RARG*, *SP7*, *PFKL*, *TRPM2*, *SUMO3*, and *TSPEAR*, exhibit promising associations with ACC based on their known functions and previous research findings. The genes *KRT71*, *KRT1*, *KRT4*, *RARG*, *PFKL*, *TSPEAR*, and *SUMO3* are involved in hair follicle formation and skin development. On the other hand, the genes *ITGB7*, *CSAD*, and *TRPM2* are associated with immune system diseases that accompany skin deficiencies. Surprisingly, both the *PFKL* and *TRPM2* genes were identified by both the 50 K chips and Imputed GWAS, further substantiating their reliability as candidate genes. Additionally, the *SP7* gene is related to skeletal developmental abnormalities in ACC.

## Discussion

The skin, the largest organ in the human body, plays a vital role in immune surveillance, wound healing, and protection against environmental challenges [22]. Skin-related diseases contribute significantly to healthcare expenditures. Extensive congenital skin aplasia, in particular, can result in neonatal mortality, while congenital skin aplasia in pig farming is associated with postpartum piglet mortality. Genetic factors have been shown to impact on many skin diseases significantly [23]. Therefore, uncovering candidate genes related to skin development and diseases can greatly facilitate diagnosing and treating of skin-related disorders.

### Candidate genes function for ACC on SSC5

Through gene function annotation, we identified seven candidate genes associated with ACC on SSC5, namely *KRT71*, *KRT1*, *KRT4*, *ITGB7*, *CSAD*, *RARG*, and *SP7*. The *KRT71* gene encodes a protein expressed in the inner root sheath of hair follicles and is implicated in disorders such as Hypotrichosis 13 and Familial Woolly Hair Syndrome (https://www.ncbi.nlm.nih.gov/gene/112802#summary). *KRT71* exhibits differential expression in various human skin regions, including the buttocks, arms, abdomen, and legs (https://www.bgee.org/gene/ENSG00000139648). Studies have highlighted the pivotal role of *KRT71* in hair formation, as it is involved in abnormal hair follicle morphology, focal hair loss, Male-pattern baldness, and abnormal hair cortex morphology [24–27]. The protein encoded by the *KRT1* gene is a member of the keratin gene family. Type II cytokeratins consist of acidic or neutral proteins that form heterotypic pairs of keratin chains and are co-expressed during the differentiation of simple and stratified epithelial tissues (https://www.ncbi.nlm.nih.gov/gene/3848). Notably, *KRT1* has been implicated in various conditions, including abnormal skin morphology, impaired skin barrier function, abnormal epidermal layer morphology, and neonatal lethality with incomplete penetrance [28, 29]. Like *KRT1*, another candidate gene, *KRT4*, encodes a protein that belongs to the keratin gene family. It has been implicated in a range of skin and hair abnormalities, including epidermal hyperplasia, abnormal morphology of the suprabasal layer in the epidermis, impaired skin barrier function, skin inflammation, abnormal morphology of the dermal layer, and diluted coat color [30–32]. All three candidate genes mentioned above are members of the keratin gene family and play crucial roles in hair and skin development. They are likely to be closely associated with the occurrence of ACC, although the specific mechanisms of their actions have not yet been elucidated.

Research findings suggest that ACC may be accompanied by developmental deficits or musculoskeletal deficiencies [10]. The candidate gene *RARG* is not only associated with skin abnormalities, such as abnormal epidermal layer morphology, abnormal epidermis stratum corneum morphology, and impaired skin barrier function [33], but also implicated in skeletal developmental anomalies, including abnormal basioccipital bone morphology, rib fusion, cervical vertebral transformation, abnormal ventral tubercle of atlas morphology, and abnormal cervical axis morphology [34–38]. The candidate gene *SP7* also correlates with skeletal developmental abnormalities [39–41]. Due to the high postpartum mortality rate in affected piglets, *CSAD* gene on SSC5, the peak SNP (5_18403003) located within, is associated with neonatal lethality and incomplete penetrance [42]. Furthermore, another candidate gene, *ITGB7*, is implicated in abnormal immune system function in animals [43, 44]. Therefore, we hypothesize that the postpartum mortality in piglets may be attributed to the actions of these two genes.

### Candidate genes function for ACC on SSC13

The *PFKL* gene on SSC13, the lead SNP (13_207176753) located within, encodes the liver (L) subunit of an enzyme that catalyzes the conversion of D-fructose 6-phosphate to D-fructose 1,6-bisphosphate, which is a crucial step in glucose metabolism (glycolysis). Previous studies have linked the *PFKL* gene to hair follicle degeneration and abnormal hair cycle catagen phase [45]. Another gene, *TRPM2*, located near another lead SNP (13_207265708), is closely associated with the innate immune system and may contribute to increased susceptibility to bacterial infections, potentially leading to mortality [46–48].

During embryonic development, the ectoderm undergoes differentiation to form the skin, hair, and skeletal system. Research has demonstrated that the *TSPEAR* gene participates in ectodermal development and plays a crucial role in tooth and hair follicle morphogenesis by regulating the Notch signaling pathway [49, 50]. It is also a candidate gene affecting wool production traits in Merino sheep [51]. The *SUMO3* gene also influences the ectoderm development in embryos, resulting in embryonic growth arrest and delayed somite formation [52]. In summary, these four strong candidate genes on SCC13 may play a crucial role in the development of ACC, ultimately leading to the manifestation of the condition in affected individuals. However, the precise mechanisms underlying their involvement require further investigation.

## Conclusion

The present study aimed to investigate the candidate genes and loci associated with ACC. GWAS analysis was conducted on a herd of 226 Duroc pigs, identifying nine significantly associated SNPs. Further investigation revealed the presence of a 483 kb QTL (5:18,196,971–18,680,098) on SSC 5 and a 159 kb QTL (13:20,713,440–207294431 bp) on SSC13. By annotating candidate genes within a 1 Mb region surrounding the significant SNPs, a total of 11 candidate genes were identified on SSC5 and 13, including *KRT71*, *KRT1*, *KRT4*, *ITGB7*, *CSAD*, *RARG*, *SP7*, *PFKL*, *TRPM2*, *SUMO3*, and *TSPEAR*. These findings shed light on the genetic underpinnings of ACC in pigs, contribute to the understanding and treatment of ACC and provide a foundation for future studies elucidating the underlying molecular mechanisms.

## Material and methods

### Ethics statement

The animals and experimental methods used in this study follow the guidelines of the Ministry of Agriculture of China and the Use Committee of South China Agricultural University (SCAU). The ethics committee of SCAU (Guangzhou, China) approved all animal experiments. The experimental animals were not anesthetized or euthanized in this study. We confirmed that all methods are reported in accordance with ARRIVE guidelines (https://arriveguidelines.org) for the reporting of animal experiments.

### Animals and phenotype

Between 2017 and 2021, a total of 108 recently farrowed (58 ACC-affected and 50 healthy) American Duroc piglets and 98 recently farrowed (48 ACC-affected and 50 healthy) Canadian Duroc piglets were collected from breeding farms in Guangdong Wen’s Foodstuffs Co., Ltd. (Guangdong, China). The farrowing sows were raised under the same environmental conditions, and all affected piglets were identified on-site personnel.

### Genotype data acquisition and quality control

The genomic DNA necessary for this study was obtained by applying the standard phenol/chloroform method to isolate and extract DNA from the tail tissues of 216 piglets. Subsequently, rigorous quality control measures were implemented, including assessment of the light absorption ratios (A260/280 and A260/230), gel electrophoresis analysis, and quantification of DNA concentration at 50 ng/μL. GenoBaits Porcine 50 K Panel was used for genotyping. After genotyping, we enhanced the genotype data to the whole-genome sequence level using an imputation strategy. We employed the Swine Imputation (SWIM) Server tool [53] with default parameter settings to perform genotype imputation, bridging the target and reference genotype data. The reference haplotype panels were constructed from whole-genome sequencing data collected from 2259 pigs, representing 44 breeds. The genotype imputation accuracy consistently demonstrated a high average concordance rate exceeding 97%, a non-reference concordance rate of 91%, and an *r*^*2*^ value of 0.89. This ensured the reliability and robustness of our imputed data. The genotype quality control of the 216 pigs was conducted by PLINK v1.9 software [54]. Individuals with a call rate of less than 90%, SNP with a call rate of less than 90% and a minor allele frequency of less than 1% were also deleted. SNP that failed the Hardy–Weinberg equilibrium test (*P* < 10^–6^) and was unmapped or located on the sex chromosome was also removed. After quality control, the final 41,362 (50 K) and 14,942,886 (SWIM imputed) SNPs are retained for subsequent analysis, respectively.

### Population structure and LD estimation

PCA and LD analysis were performed using the SNPs that passed quality control standards to investigate the population structure of the two Duroc pig populations. PCA was performed with GCTA software v1.92.4beta [55]. The average LD decay distance across the whole genome of the American and Canadian Duroc pigs was calculated using PopLDdecay software [56].

### Single-locus GWAS analysis

In the present study, the GEMMA software v0.98.1 [21] was used to implement MLM for single-locus GWAS of ACC. GEMMA calculated the genomic relatedness matrix (GRM) between individuals within each population to account for the population structure. The first five principal components calculated by GCTA tool [55] are embedded into the MLM as covariables to eliminate the mixed influence of population structure. The MLM is as follows:

$$\mathbf{y}=\mathbf{W}{\varvec{\upalpha}}+\mathbf{X}{\varvec{\upbeta}}+\mathbf{u}+{\varvec{\upvarepsilon}}$$

where **y** represents a vector of the phenotype, with affected piglets assigned as 1 and healthy individuals assigned as 0; ***W*** is the incidence matrix of covariates, including fixed effects of the sex, and the top five principal components from PCA analysis; α represents the vector of corresponding coefficients including the intercept; ***X*** is the vector of all marker genotypes; **β** specifies the corresponding effect size of the marker; u is the vector of random effects, with ***u* ~ MVN**_**n**_** (0, λτ**^**−1**^** K)**; **ε** is the vector of random residuals, with **ε ~ MVN**_**n**_** (0, τ**^**−1**^*** I***_***n***_**)**; **λ** signifies the ratio between two variance components;** τ**^**−1**^is the variance of the residual errors; ***K*** is GRM; *I* is an n × n identity matrix and n refers to the number of pigs. In the 50 K chips GWAS, the Bonferroni method was used to determine the genome-wide significant threshold (0.05/N), where N is the number of SNPs. Since that is a stringent criterion, we set a more lenient threshold to detecting the suggestive SNPs (1/N) [20, 57]. Furthermore, to address the potential type I errors introduced by population stratification, we employed the GMMAT R package to perform logistic mixed model GWAS specific to binary traits [58]. Based on human GWAS results, we set the genome-wide significance threshold and suggestive significance threshold for GWAS based on imputed data at 5.00E-8 and 1.00E-6, respectively [59, 60].

### Haplotype block analysis and conditional analysis

The PLINK v1.9 [54] and Haploview v4.2 [61] were used for chromosomal regions with multiple significantly clustered around the top SNP to calculate the LD pattern of the region. GWAS often identifies a significant set of SNPs associated with target traits in putative regions, possibly due to high LD between them. Conditional analysis was conducted by fitting the genotypes of peak SNP as covariates to the univariate MLM of GEMMA to evaluate the independence of the signal peak in the putative region.

### Identification of candidate genes and functional enrichment analysis

Based on the LD decay distances of the American and Canadian Duroc populations, the candidate genomic regions were determined to 500 kb on either side of the significant SNPs. All SNPs refer to the latest version of the *Sus scrofa* 11.1 genome (http://ensembl.org/Sus_scrofa/Info/Index). Functional gene annotation (v105) was downloaded in GIFF3 format from the Ensembl website (http://ftp.ensembl.org/pub/release-105/gff3/sus_scrofa/). The R package BioMart [62] efficiently retrieved functional genes. KEGG [63] and GO analyses were conducted using KOBAS 3.0 [64] to investigate the functions of all candidate genes. Enriched terms with a significance threshold of *P* value < 0.05 were selected to explore further the genes invoked in pathway and biological processes [19]. Subsequently, we employed REVIGO (http://revigo.irb.hr) in conjunction with the Mus musculus database to eliminate GO term redundancy (medium threshold, 0.7) and cluster the remaining terms in a 2D space [65, 66]. This space was derived by applying multidimensional scaling to a matrix of GO term semantic similarities. Mouse Genome Informatics website (https://www.informatics.jax.org/), GeneCards (http://www.genecards.org/) and Ensembl (www.ensembl.org/biomart/martview) were used to query gene functions.

### Supplementary Information

**Additional file 1: Fig S1.** Photos of ACC-afflicted piglets.

**Additional file 2: Table S1. **Distributions of SNPs after QC and the average SNPs on each chromosome for ACC trait.

**Additional file 3: Fig S2. **Manhattan plots of Imputed GWAS for ACC in 216 pigs. Description: The x-axis represents the chromosomes, and the y-axis represents the -log10(*P*-value). The dashed lines indicate the thresholds for ACC (*P* =1.00E-6).

**Additional file 4: Table S2. **Significant SNPs associated with ACC in Duroc pigs identified by imputed GWAS. Description: This file provides information about significant SNPs discovered in the GWAS with imputed data.

**Additional file 5: Fig S3. **Manhattan plots of 50K GWAS using logistic mixed model for ACC in 216 pigs. Description: The x-axis represents the chromosomes, and the y-axis represents the -log10(*P*-value). The dashed lines indicate the suggestive thresholds for ACC (*P* =2.42E-5).

**Additional file 6: Table S3. **Genes annotation within 1 Mb surrounding nine significant SNPs identified in GWAS. Description: This file provides the positional information of protein-coding genes within a 1 Mb region surrounding significant SNPs.

**Additional file 7: Table S4. **Pathway Enrichment Analysis Reveals Significantly Enriched KEGG and GO Pathways Related to ACC. Description: This file provides the enrichment of protein-coding genes in KEGG and GO terms (*P* < 0.05).

## Data Availability

The SNP genotyping data presented in this study are deposited in the figshare (https://figshare.com/articles/dataset/Duroc_ACC_50K_vcf_gz/24346768).

## Abbreviations

ACC: Aplasia cutis congenita
SSC: *Sus* *scrofa* Chromosome
GWAS: Genome-wide association study
SNP: Single nucleotide polymorphism
QTL: Quantitative trait locus
LD: Linkage disequilibrium
MLM: Mixed linear model
PCA: Principal component analysis
KEGG: Kyoto Encyclopedia of Genes and Genomes
GO: Gene Ontology
