# Supplementary figures and images for: Genome-wide association analysis unveils candidate genes and loci associated with aplasia cutis congenita in pigs
Source: BMC Genomics. 2023 Nov 21;24:701. doi: 10.1186/s12864-023-09803-6 (PMC10664689; doi:10.1186/s12864-023-09803-6)

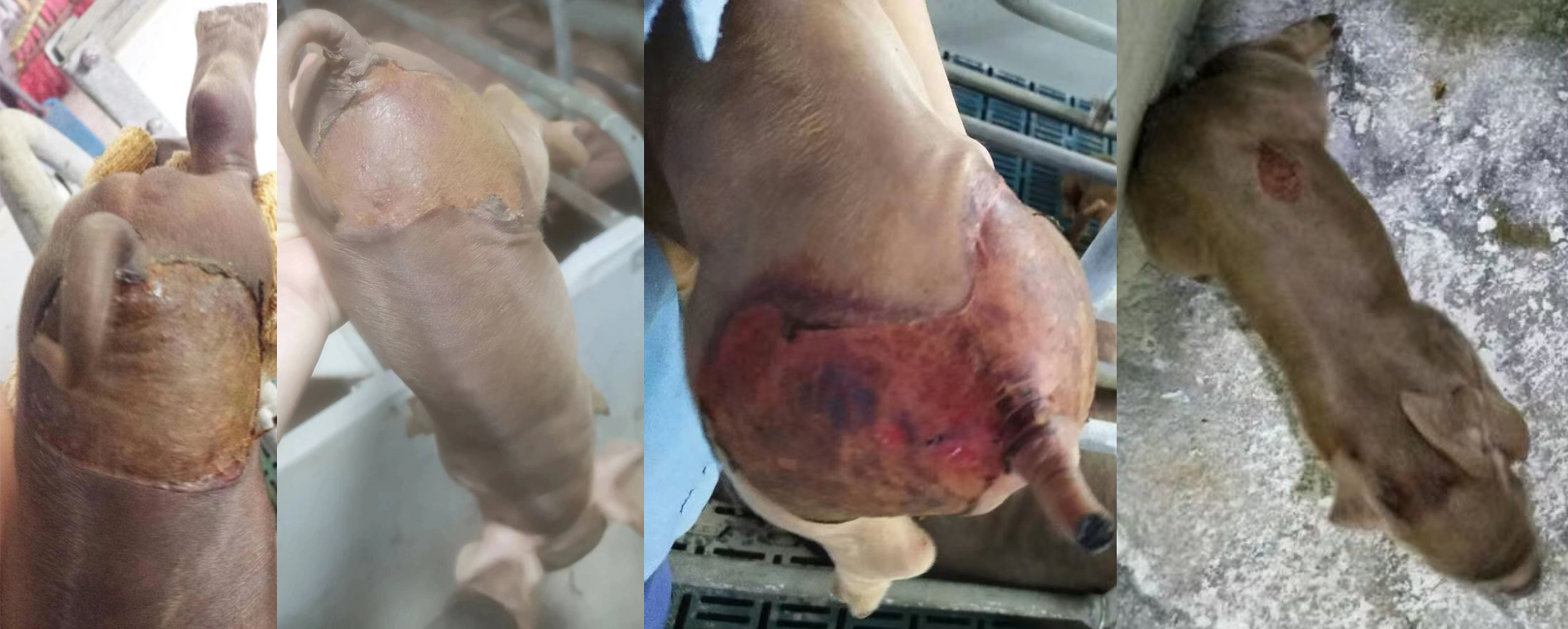

Supplement: Supplementary file 1 — Additional file 1: Fig S1. Photos of ACC-afflicted piglets. [file 12864_2023_9803_MOESM1_ESM.png]

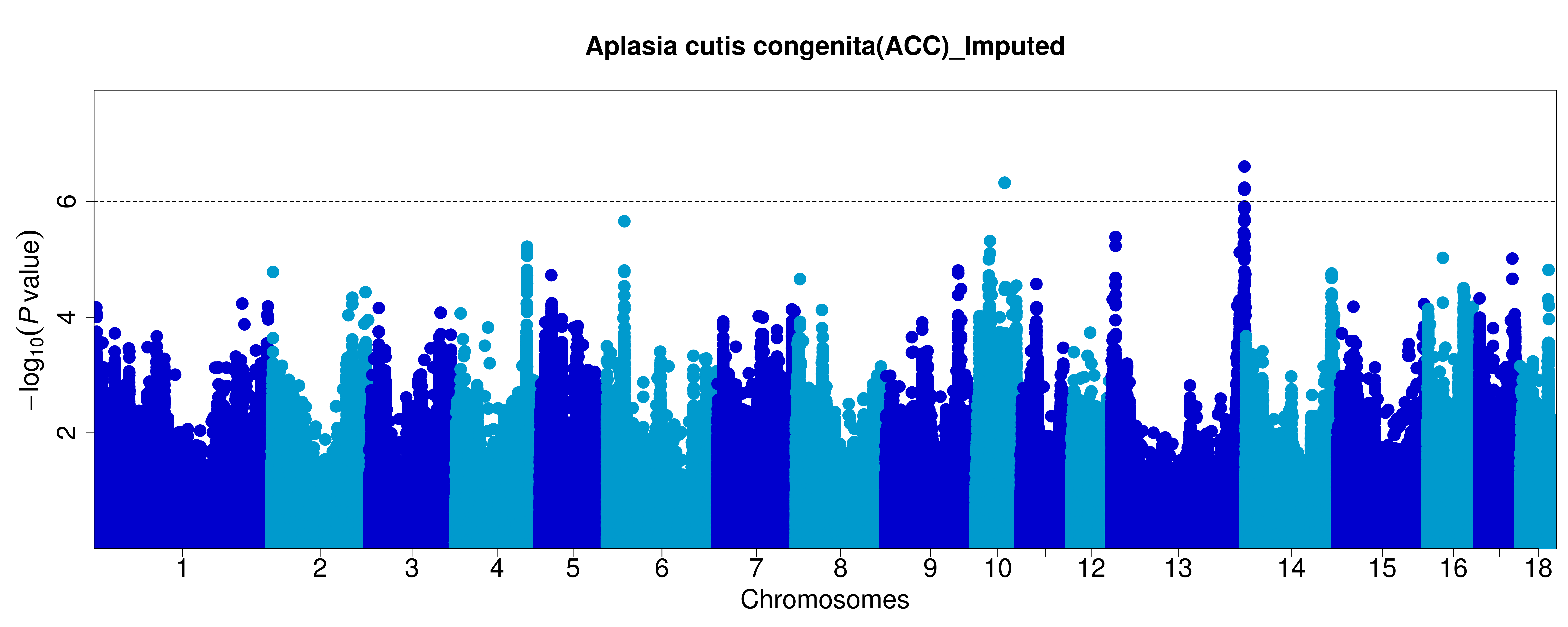

Supplement: Supplementary file 3 — Additional file 3: Fig S2. Manhattan plots of Imputed GWAS for ACC in 216 pigs. Description: The x-axis represents the chromosomes, and the y-axis represents the -log10(P-value). The dashed lines indicate the thresholds for ACC (P =1.00E-6). [file 12864_2023_9803_MOESM3_ESM.png]

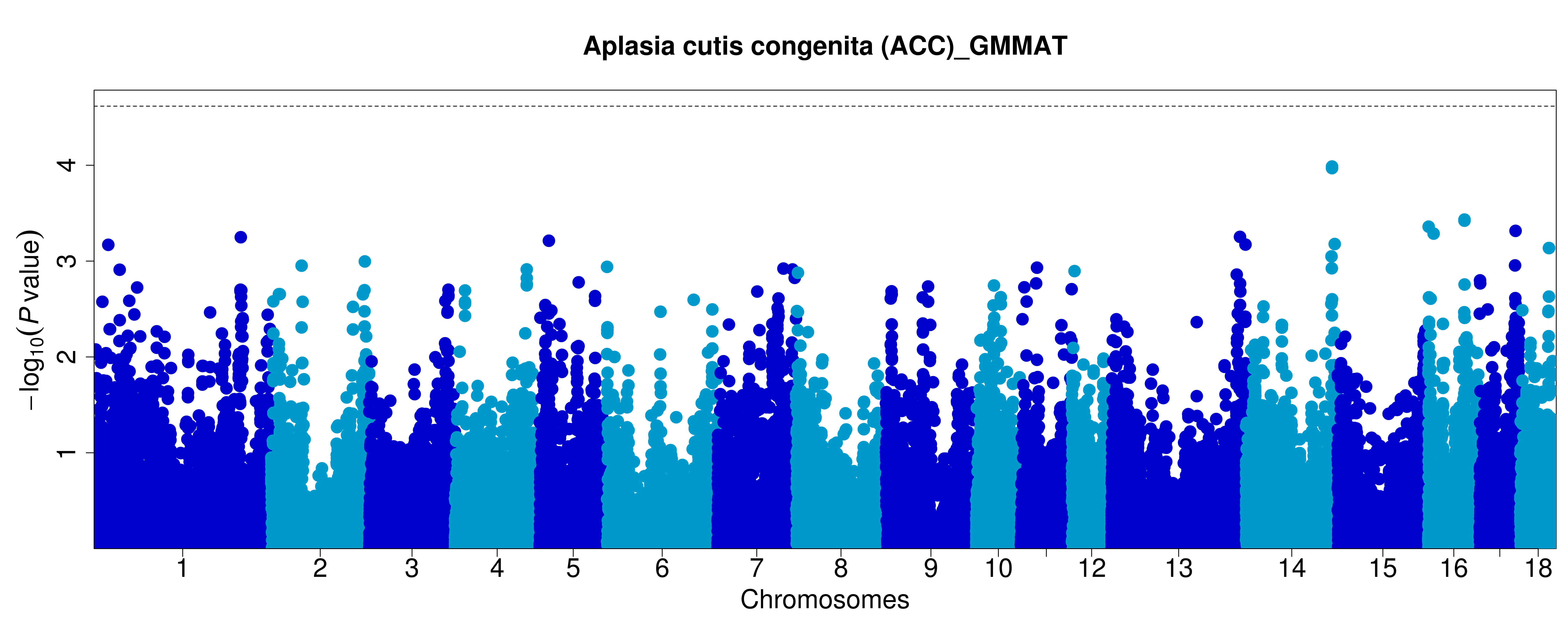

Supplement: Supplementary file 5 — Additional file 5: Fig S3. Manhattan plots of 50K GWAS using logistic mixed model for ACC in 216 pigs. Description: The x-axis represents the chromosomes, and the y-axis represents the -log10(P-value). The dashed lines indicate the suggestive thresholds for ACC (P =2.42E-5). [file 12864_2023_9803_MOESM5_ESM.png]
